# Supplementary material for: Reduced cellular binding affinity has profoundly different impacts on the spread of distinct poxviruses
Source: PLoS One. 2020 Apr 30;15(4):e0231977. doi: 10.1371/journal.pone.0231977 (PMC7192435; doi:10.1371/journal.pone.0231977)
Supplement: S1 Fig — Shown are regions drawn around individual VACV plaques 48 hour after infection of the indicated cells (region shown as white circle) given as examples of how foci/plaque size was determined. Foci area calculated for each region using ImageJ is shown below image. Note that these specific images/regions are presented only as after the fact examples of how regions were drawn. The specific visual images of the regions drawn for data acquisition were not saved in ImageJ and therefore only the calculated areas for each foci/plaque remain. (DOCX) [file pone.0231977.s001.docx]

**
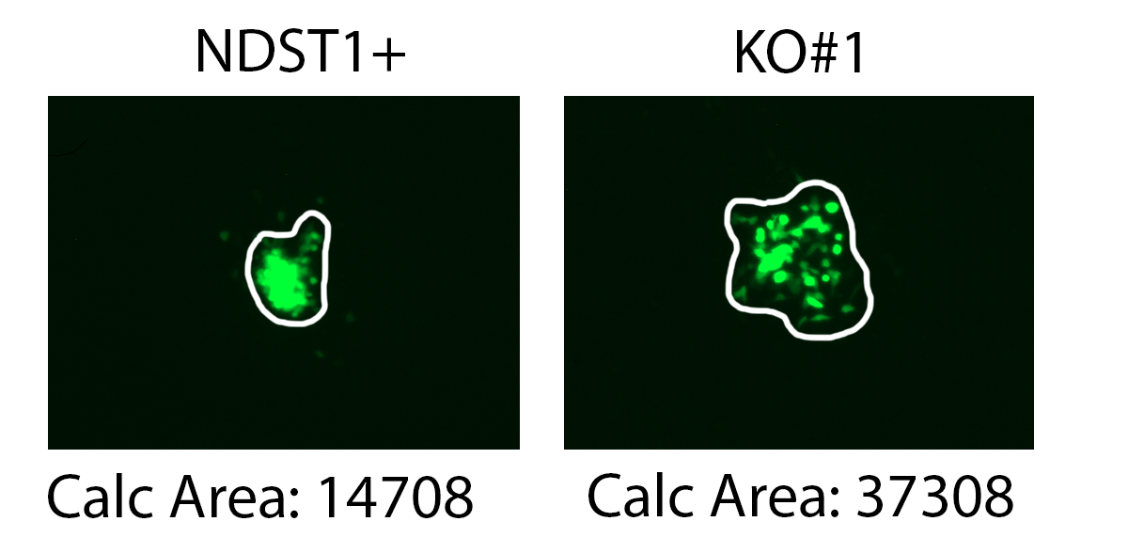
**

**Supplemental Figure S1: Examples of foci/plaque quantitation:** Shown are regions drawn around individual VACV plaques 48 hour after infection of the indicated cells (region shown as white circle) given as examples of how foci/plaque size was determined. Foci area calculated for each region using ImageJ is shown below image. Note that these specific images/regions are presented only as after the fact examples of how regions were drawn. The specific visual images of the regions drawn for data acquisition were not saved in ImageJ and therefore only the calculated areas for each foci/plaque remain.
