# Supplementary material for: Reduced cellular binding affinity has profoundly different impacts on the spread of distinct poxviruses
Source: PLoS One. 2020 Apr 30;15(4):e0231977. doi: 10.1371/journal.pone.0231977 (PMC7192435; doi:10.1371/journal.pone.0231977)
Supplement: S2 Fig — mRNA was extracted from untreated wild-type B16/F10 cells and used to synthesizes cDNA. Two different primer sets (corresponding to each previously reported NDST enzyme—NDST1, NDST2, NDST3, and NDST4) were then used to attempt to amplify regions of each gene from the cDNA. Successful PCR amplification was observed with both primer sets corresponding to NDST1 and NDST2. No specific PCR products were observed in either primer set against NDST3 or NDST4. Note that due to low technical quality the image shown has been enhanced for both brightness and contrast as well as cropped to remove irrelevant lanes on the right side. (DOCX) [file pone.0231977.s002.docx]

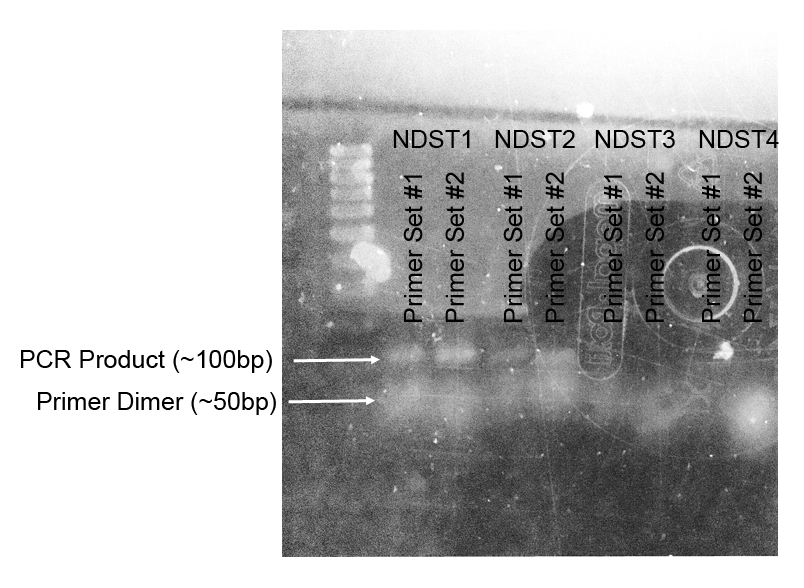


**Supplemental Figure S2: Expression of NDST1-4 in B16/F10 cells**. mRNA was extracted from untreated wild-type B16/F10 cells and used to synthesizes cDNA. Two different primer sets (corresponding to each previously reported NDST enzyme - NDST1, NDST2, NDST3, and NDST4) were then used to attempt to amplify regions of each gene from the cDNA. Successful PCR amplification was observed with both primer sets corresponding to NDST1 and NDST2. No specific PCR products were observed in either primer set against NDST3 or NDST4. Note that due to low technical quality the image shown has been enhanced for both brightness and contrast as well as cropped to remove irrelevant lanes on the right side.
