# Supplementary material for: Reduced cellular binding affinity has profoundly different impacts on the spread of distinct poxviruses
Source: PLoS One. 2020 Apr 30;15(4):e0231977. doi: 10.1371/journal.pone.0231977 (PMC7192435; doi:10.1371/journal.pone.0231977)
Supplement: S3 Fig — Original scan of NDST western blot used in Fig 1B. (PDF) [file pone.0231977.s003.pdf]

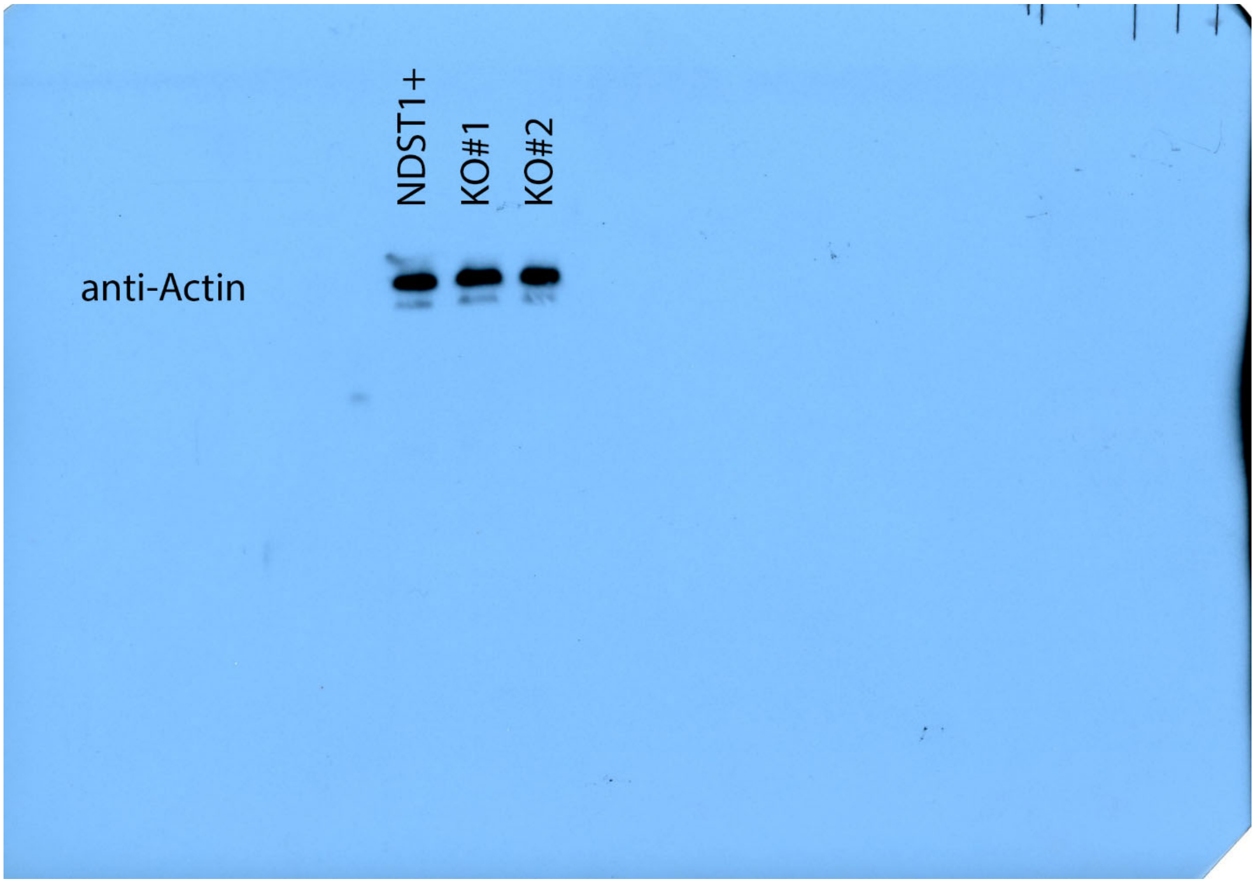

Supplemental Figure S3: Original scan of Actin western blot used in Fig 1B.

Note that figure has been labeled for clarity but has not been altered in any other way.

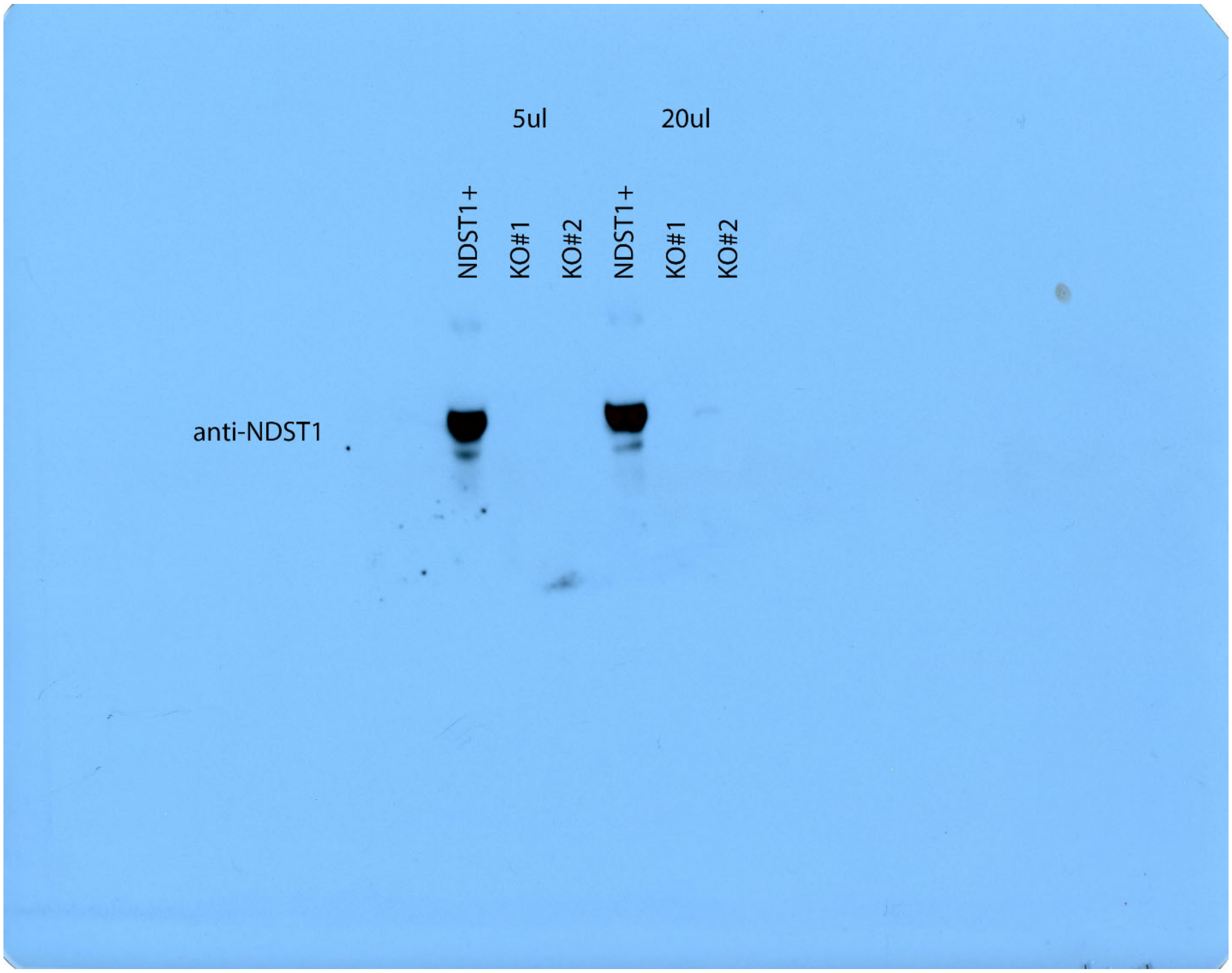

Supplemental Figure S3: Original scan of NDST western blot used in Fig 1B.

Note that figure has been labeled for clarity but has not been altered in any other way.
