# Supplementary material for: Reduced cellular binding affinity has profoundly different impacts on the spread of distinct poxviruses
Source: PLoS One. 2020 Apr 30;15(4):e0231977. doi: 10.1371/journal.pone.0231977 (PMC7192435; doi:10.1371/journal.pone.0231977)
Supplement: Attachment — Submitted filename: Response to Editorial Comments.pdf [file pone.0231977.s005.pdf]

Additional Editor Comments (if provided):

Thank you very much for paying so close attention to all the reviewers and addressing all issues raised by both reviewers. The manuscript has been improved as a result. However, a couple of issues still need some attention, as listed below.

Please see responses to specific comments below. Note that no changes needed to be made to the figures based on these comments.

1. Statistics. A few figures show error bars, which are described as SD, and present p values, but are described as presenting the sum of two independent experiments (figure 2B, 3A, 4), or the number of experiments is not presented (figure 5). SD and p values using Student's T test or similar require a minimum of 3 independent experiments to test the biological reproducibility of a result. Please clarify what are the error bars and p values. If only two or one biologically independent experiments are presented, then SD and p values cannot be calculated.

We apologize for the confusion. The references in the figure legends to two independent experiments refers to completely separate experiments done on different days. Each experiment was conducted in at least triplicate giving us a total n of 6+. Stats are calculated for the total n which actually gives us relatively solid statistical power. We have amended the figure legends to make this clearer.

2. Wording. "Semi-quantitative", although used in biology, is actually a self-contradictory word: an approach is by definition quantitative or qualitative. In this particular paper, the word does not add much, and it can well be removed (line 178)

We totally agree with this assessment. We included the semi-quantitative phrase, simply because that is typically how agarose gels are referred to. This has now been replaced with 'Qualitative" as requested.

3. Clarity to the reader. Although discussed in the discussion section, the presentation of Figure 4 in the results appears contradictory to the results from figure 3. Perhaps you may want to anticipate the likely explanation in the results section, and then present, as you do now, the full discussion in the discussion section? I am of the opinion that it would be helpful to the reader.

We have now included the following phrase in the results section describing Fig 4. "Indeed, overall VACV infection in this experiment was inefficient compared to other previous infections (Fig 3) which is likely due to minor experimental variation." Combined with the previously included section about this issue in the discussion we hope that this provides sufficient clarity to the reader concerning the observed results.

4. Lastly, although the discussion about the effects of MC is now far more clear, there is an issue that is not actually discussed. The effect adding MC on VACV plaque size is not by decreasing the size of the plaques in the knockout cells, but rather by increasing the size in the wild-type ones. This may perhaps be a result of experimental variability, but it does not appear to affect the size of MYXV. This unexpected observation deserves some discussion, even it were just to state that it is an intrinsic experimental variability.

You had to go there. Unlike the data in Figs 3 and 4, this phenotype is highly reproducible suggesting it is not due to experimental variation. We did not discuss it in the previous version of the manuscript since we don’t have a true explanation for it. Our working hypothesis is that this phenotype is the result of MC trapping viral particles close to the site of initial infection thus increasing the local concentration of virus (resulting in more infection). This is actually fairly consistent with our overall model which proposed that NDST1 deficiency causes reduced binding efficacy effectively spreading out VACV infections. While this is only hypothetical, I have added a new paragraph into the discussion regarding this possibility (note that I also had to rearrange some of the surrounding sentences to make the grammer flow properly).
