# Supplementary material for: Reduced cellular binding affinity has profoundly different impacts on the spread of distinct poxviruses
Source: PLoS One. 2020 Apr 30;15(4):e0231977. doi: 10.1371/journal.pone.0231977 (PMC7192435; doi:10.1371/journal.pone.0231977)
Supplement: Attachment — Submitted filename: Response to Reviewre Comments.docx [file pone.0231977.s006.docx]

**Comment from Reviewer 3:**

**“…Therefore, it is correct to treat them as independent and to combine them to obtain the final estimate and use statistical tests for independent statistical units such as the Student t-test. My only suggestion is that because of the small sample size the authors should consider using a non-parametric test instead of the Student T-test which relies on the distribution of the data be approximately normal.”**

Based on the statistical review of our previous data analysis, it appears that our previously utilized approach of combining multiple experiments is statistically valid. As requested, we have reanalyzed the combined data set using the non-parametric Mann-Whitney test (which also yields significance for the data) and adjusted the figure legend and figure accordingly.
