# Supplementary material for: Reduced cellular binding affinity has profoundly different impacts on the spread of distinct poxviruses
Source: PLoS One. 2020 Apr 30;15(4):e0231977. doi: 10.1371/journal.pone.0231977 (PMC7192435; doi:10.1371/journal.pone.0231977)
Supplement: Attachment — Submitted filename: Response to Reviewer Comments.docx [file pone.0231977.s004.docx]

**Response to Reviewer Comments**

**Editorial Comments:**

1) **Please ensure that your manuscript meets PLOS ONE's style requirements**

We have edited the manuscript for style to conform to Plos One’s requirements (apologies). Note that these changes are not tracked on the marked up file.

2) **PLOS ONE now requires that authors provide the original uncropped and unadjusted images underlying all blot or gel results**

Our manuscript only contains 1 figure with western blot data (Fig 1B). The original files for the scanned in gels have now been included as supplemental figures 3 and 4. The scanned in images have been labeled for clarity but not altered in any other way. Note that these supplemental files are not referenced in the manuscript.

3) **Please include your tables as part of your main manuscript and remove the individual files.**

Table 1 has now been inserted into the manuscript file as requested.

**Reviewer #1**

**1) Much of the discussion hinges on the measurement of "plaque size", but how this was determined by the authors is not clearly explained. This is actually quite tricky judging by the images shown in Fig5C or 6A, since there aren't clear margins to the plaques. Is it a measure of the diameter from the outermost of the GFP+ pixels or is it perhaps the area enclosing some % of the GFP+ signal? Some clarity here would be helpful.**

We apologize for the lack of clarity. The reviewer is correct in their assumption that plaque size was determined by drawing an area around the outermost ring of GFP+ cells and subsequently determining the area of that region using post image processing (we used ImageJ for this). This is an approach that we have published on previously and we therefore did not include much discussion of this as we simply cited our previous work. We have now removed that citation and instead included a more complete methods section on measuring foci/plaque size. Examples of how we drew the regions around foci are also now included as supplemental Figure S1.

**2) The qPCR data in Fig 4B are odd. Given the discrepant measurements at 10 and 12 hr (in cell line #1 and 2), it's very difficult to come to any conclusions regarding whether DNA synthesis is affected by this mutation. This experiment should be repeated. Ideally one would like to know whether the replication kinetics are any different once the barrier to infection has been overcome (i.e post entry). That means identifying newly infected cells and measuring DNA synthesis in just those cells. Is there any way to determining this? For example, could one measure the rate of growth of DAPI strained virus factories once they are first detected?**

We fully agree with the reviewer that our genome quantitation data should have been repeated. We have therefore repeated the previous experiment two additional times as well as also conducted a similar experiment for VACV (which was not previously shown) three separate times. The summation of all three experiments is now shown as new data in Fig 4B. Interestingly, all three replicates for MYXV do show the previously observed decrease in genomes between 10 and 12 hours post infection. While we do not have an explanation for this phenomenon, we hypothesize that it could be due to cellular lysis during this time frame. We would also point out that it occurs in both control and NDST1 deficient cells and is therefore not likely to impact the conclusions of our work.

**3) Lines 251-62. The paragraph surrounding the role of EEV is confusing. The authors lead with a statement that MYX produce much less EEV than VAC, then use methyl cellulose to "inhibit release of EEV", see no effect of methyl cellulose on VAC plating in either cell type, and conclude that EEV play a major role in determining VAC spread. I can't follow the logic, there seems to be a non sequitur or circular reasoning somewhere. As an aside, VAC strain WR doesn't produce much EEV, the effect seen here might be more striking if strain IHD-W was used.**

We apologize for the lack of clarity. The phenotype under study was the increased size of VACV plaques seen in NDST1 deficient cells (Fig 5). The data in figure 6 shows that this phenotype goes away in the presence of MC and that under these conditions VACV forms identical sized plaques in both NDST+ and deficient cells. The effect of MC is therefore that it eliminates the previously observed phenotype. The corresponding results section has been edited to hopefully clarify this point.

**4) Lastly, one is still left uncertain whether this mutation is affecting binding alone or binding plus entry via the entry fusion complex. Fig 2 doesn't really help clarify this point and it's only briefly discussed in lines 296 - 300. Have the authors tried to measure a time lag between binding (for example on ice) and uncoating?**

We have not directly measured the lag between binding and entry/uncoating. However, our data in Fig 4A measures early gene expression following high MOI infection which is similar to the requested experiments (albeit a slightly more indirect measure). This data clearly shows a delay in GFP expression in NDST1 deficient cells for both VACV and MYXV. We interpreted this as a possible delay in entry in our discussion, however, we could not rule out the possibility that GFP expression in these experiments is virion dose dependent. We have edited the relevant section in the discussion to further emphasize this point.

**5) Abbreviations need to be explained (e.g. NTC)**

We apologize for this oversight. We have re-read the manuscript and now provide definitions for all abbreviations used in both the text and the figures upon their first usage.

**6) The green plaque images (e.g. Fig 5A) would be better presented as black on white as in Fig 5C.**

We have replaced the images of green foci/plaques in both Fig 5 and Fig 6 with inverted black and white images as requested.

**7) There's an NDST2 I gather, would it still be functional?**

These are actually 4 distinct NDST enzymes (NDST1-4). We have now tested expression of all of these enzymes in B16/F10 cells and find expression of NDST1 and 2 (but not 3 and 4). This data is now included as supplemental Figure S2. However, of these four enzymes, NDST1 is absolutely essential for heparin sulfation and this cannot be overcome by the presence of NDST2-4 in the absence of NDST1. Therefore, our KO of NDST1 efficiently prevents all heparin sulfation (Fig 1E) even though NDST2 should still be present in our KO cells (note that we have not directly tested expression of NDST2 in our NDST1 KO cells). A sentence has been added to the results section (line 143) to clarify this.

**8) Can one call NDST1 "essential" as the authors do in the abstract? The k/o cells seem to grow fine.**

We apologize for the lack of clarity. NDST1 is not essential for cell growth, however, it is completely essential for GAG sulfation (which is what we were attempting to refer to here). We have edited the referenced section of the abstract to clarify this point.

**9) As an experiment for another day I would be curious to know if virus grown on k/o cells have any subsequent problems infecting normal cells? Presumably the progeny aren't sulfated properly.**

This is a very interesting point that we had not initially considered! One would presume that some proteins within the poxviral virion are heparinated and therefore most likely sulfated as well and that this might play a role in poxviral binding or entry (based on the charges involved it might actually be a negative binding regulator). This could probably be examined be looking at the particle:PFU ratios of virions grown in NDST1+ and deficient cells, however, as noted by the reviewer, this likely falls outside the scope of the current manuscript. We’ve added a new paragraph into the discussion section on this issue.

**Reviewer #2**

**1. Can the authors explain the discrepancy in the results between Fig. 3A and Fig. 4A? In Fig. 3A, where viral GFP expression is measured at 24 hours post infection, ~100% of cells are infected regardless of NDST1 deletion at a high MOI (i.e., 3). However, in Fig. 4A, where cells were also infected at a high MOI (i.e., 10), but at 24 hours, only ~20% of NDST1-deleted cells were GFP+ compared to 100% in the NDST+ cells.**

We assume that the review is referring to the VACV results in these figures as the MYXV results appear fairly consistent. Regarding the VACV infection, we completely agree with the reviewer that the results of the two experiments are slightly different. We have attribute these differences to slight experimental variation. Overall infection with VACV in the experiment shown in Fig 4 was inefficient compared to that seen in Fig 3 (Note that the rate of infection at 24 hours in the control NDST1+ cells seen in Fig 4 is actually only 71% which is significantly lower than the 91% seen in Fig 3). While we do not have a concrete explanation for these experimental changes, we would note that poxviral infection is highly dependent on both concentration and the number of freeze thaws a specific stock has undergone (both variables which are difficult to get completely identical over the course of multiple experiments). Regardless of these differences, we do feel that we have included the appropriate control groups in both Fig 3 and Fig 4 and that the results of these groups (while not identical) are within the realm of biological variation and result in fairly consistent conclusions. We therefore feel that the conclusions drawn from these results remain valid. We have included a sentence in the results section (line 202-203) indicating that we believe these differences are due to minor biological variations across experiments.

**2. Figure 4B: The data would be better expressed as genome copies (based on comparison to a standard curve). Furthermore, the data are from one experiment. I would suggest repeating the experiment and showing data from three independent experiments, particularly considering the discrepancies observed between the two NDST1-deleted cell lines. In particular, the phenotype in NDST1-deleted cell line #1 appears strange, with viral genomes dropping between 10 and 12 hours.**

Replication of our real time PCR quantitation of viral genomes has been addressed in reviewer #1’s comment #2 above.

We have previously considered the possibility of generating standard genome curves to present our data as genomes copies. Unfortunately, our experience is that using whole virions for a standard is not accurate due to poor PCR efficiency from intact virus particles (compared to free viral genomes within a cell) and extraction of viral DNA from infected cells yields high levels of cellular genome contamination. We therefore feel that our use of differential CT values represents the most accurate method to display this data.

**3. Why were B16/F10 cells chosen for this study? A brief justification of the cell line chose may be helpful for non-experts. Furthermore, BSC40 cells appear to have been used in Figure 4 (as indicated on line 379). Why the change?**

The reviewer raises an excellent point concerning the origination of our study and the reason why B16/F10 cells were chosen. In full disclosure, our lab is primarily interested in oncolytic immunotherapy. In this context, B16/F10 cells represent a standard model for melanoma tumors which represent one of the major targets for virotherapy. The original purpose of removing NDST1 from these cells was to study the role of susceptibility to viral infection during oncolytic treatment (note that a separate manuscript using the NDST1-/- cells in this context is current under preparation). In the process of doing this oncolytic work, we kind of stumbled upon the differential effects of NDST1 loss on MYXV and VACV infection. Due to this background, there is really not a strong scientific rationale for undertaking the basic virology aspect of this work in B16/F10 cells. However, despite the poor initial justification for the cell type used, we do feel that the work remains scientifically strong and that the results obtained are interesting enough to warrant publication. Note that I’m not really sure how to clarify this point coherently in the actual manuscript and so no changes have been made in the text regarding this particular issue.

The reference to BSC40 cells on line 379 was a typo and has been corrected to refer to NDST1+ or deficient B16/F10 cells.

**4. To confirm that the phenotypes observed are indeed due to the absence of NDST1 (and not clonal selection during cell line construction), I would suggest adding back NDST1 to confirm the effect is reversed by NDST1 expression. Similarly, do the authors see the same phenotype (reduced spread of MYXV, but enhanced spread of VACV) with heparase treatment?**

We agree with the reviewer that reconstitution experiments are one method to demonstrate the specificity of CRISPR-based genetic mutations. However, these reconstitution experiments are often not trivial since overexpression comes with numerous caveats. This is one of the major reasons that these types of experiments are rarely performed in any study using CRISPR. In the absence of this type of data, we would argue that both our NDST1 KO cells lines are clonally derived and display identical phenotypes in basically all of our proposed experiments. The chances of this occurring form an off-target, CRISPR mutation in two distinct cell lines is extremely small. Additionally, the phenotypes observed in our studies are highly consistent with previous work on the biology of NDST1 and poxviruses. We therefore feel confident that our results are due to specific loss of NDST1.

**5. The authors nicely explain in the introduction that VACV binds to both heparan sulfate and chondroitin sulfate. Is the reduced effect of NDST1 deletion on VACV compared to MYXV due to its binding to CS? And similarly is the enhanced spread of VACV in the absence of NDST1 facilitated by binding to CS in distant cells? It would be interesting to evaluate the effect of CS removal (i.e., by chondroitinase treatment) in the NDST1 deleted cells, to see whether the VACV phenotype becomes more like the MYXV phenotype.**

We agree that the possibility of VACV having a higher binding affinity to CS could play a role in our observed outcomes. Unfortunately, we are not aware of any genetic methodology to specifically eliminate sulfation on CS and genetically eliminating chondroitin itself is likely to have widespread effects which would be difficult to interpret. It would likely be possible to partially overcome this with enzymatic digestion (chondroitinase), however, it’s a fairly large amount of work to do this type of study well and we therefore feel that it is outside the scope of the current manuscript. A sentence has been added to the discussion section concerning the possible role of CS in VACV binding.

**6. Line 248: “context” should be “content”**

This typo has been corrected

**7. Line 315: “typical amounts EEV” should be “typical amounts of EEV”**

This typo has been corrected

**8. Line 378: “kinetic” should be capitalized to read “Kinetic”**

This typo has been corrected
